# Risk Factors, Clinical Characteristics, Management, and Outcomes of Musculoskeletal Fungal Infection at Thailand’s Largest National Tertiary Referral Center

**DOI:** 10.3390/jof8020191

**Published:** 2022-02-16

**Authors:** Piyaporn Chokevittaya, Methee Chayakulkeeree, Wanruchada Katchamart

**Affiliations:** 1 Department of Medicine, Faculty of Medicine Siriraj Hospital, Mahidol University, Bangkok 10700, Thailand; chokevittaya.4th@gmail.com; 2 Division of Infectious Diseases and Tropical Medicine, Department of Medicine, Faculty of Medicine Siriraj Hospital, Mahidol University, Bangkok 10700, Thailand; mcha8715@yahoo.com; 3 Division of Rheumatology, Department of Medicine, Faculty of Medicine Siriraj Hospital, Mahidol University, Bangkok 10700, Thailand

**Keywords:** risk factors, clinical characteristics, management, outcomes, musculoskeletal fungal infection, osteomyelitis, septic arthritis

## Abstract

To investigate the risk factors, clinical characteristics, management, and outcomes of musculoskeletal fungal infection in Thai patients, patients aged ≥18 years definitively diagnosed with musculoskeletal fungal infection by culture and/or histopathology at Siriraj Hospital (Bangkok, Thailand) during 2002–2020 were retrospectively enrolled. Twenty-eight patients (median age: 58.5 years [range: 22–81], 57.1% male) with fungal osteomyelitis (n = 22), septic arthritis (n = 1), or fungal osteomyelitis with septic arthritis (n = 5) were included. Immunocompromised status was common (82%). Most patients had de novo infection from hematogenous spreading that usually presented at a single, non-contiguous site. The median symptom duration prior to diagnosis was 2 months. The tibia and knee were the most common site of osteomyelitis (30%) and septic arthritis (72%), respectively. The most common pathogens were *Talaromyces marneffei* and *Cryptococcus neoformans.* Organism identification from tissues at the affected sites was required in all cases. Most patients (82%) required combination surgery and systemic antifungal therapy. Among those with complete follow-up (23/28), 61% and 39% had complete and partial responses, respectively. Musculoskeletal fungal infection is an uncommon disease with insidious onset and non-specific manifestations that requires pathogen identification via tissue cultures and histopathologic studies. Combination surgery and systemic antifungal therapy yielded generally favorable outcomes.

## 1. Introduction

Infection of the bone, joint, and/or muscle is a common medical condition. Patients commonly present with the clinical signs/symptoms of warmth, swelling, tenderness, and/or limited range of motion at the affected area. The clinical varieties of these infections include osteomyelitis, septic arthritis, myositis, tendinitis, enthesitis, and bursitis [1].

For septic arthritis, three mechanisms of infection have been proposed, including hematogenous spreading, direct inoculation, and contiguous infection from an adjacent area [2]. The common pathogens that cause septic arthritis are bacteria, such as *Staphylococcus* spp., *Streptococcus* spp., and *Mycobacterium* spp.—especially in endemic areas. Septic arthritis that is caused by pathogens other than bacteria, such as fungi, is relatively rare.

The pathogens that most commonly cause musculoskeletal fungal infection include *Candida* spp., *Aspergillus* spp., *Coccidioides* spp., *Histoplasma* spp., *Cryptococcus* spp., and *Blastomyces* spp. Other fungi that have been reported as potential causative pathogens in musculoskeletal fungal infection include *Sporothrix schenckii, Acremonium* spp., *Scedosporium* spp., *Peteriellidum boydii*, and *Trichophyton rybrum* [3,4,5].

Musculoskeletal fungal infections, especially osteomyelitis, commonly occur in immunocompromised hosts and tend to be insidious in process with non-specific clinical manifestations. Therefore, they are often associated with significant morbidity and/or mortality due to delayed diagnosis and improper management. Therefore, early diagnosis and aggressive treatment are crucial for achieving a favorable outcome.

There have been various case reports and case series describing musculoskeletal fungal infection; however, data from Asia, including Thailand, are scarce [3]. Accordingly, we conducted this study to improve our understanding of the epidemiology, clinical risk factors, clinical manifestations, diagnosis, management, and outcomes of musculoskeletal fungal infection in Thailand.

## 2. Materials and Methods

Patients aged ≥18 years who were definitively diagnosed with musculoskeletal fungal infection by culture and/or histopathology at the Faculty of Medicine Siriraj Hospital, Mahidol University, Bangkok, Thailand, during 2002–2020 were retrospectively enrolled. Definite diagnosis of musculoskeletal fungal infection was defined as the identification of organisms from the synovial fluid, pus, or discharge from affected areas or from the bone, muscle, tendon, ligament, synovium, or bursa using at least one of following methods: direct microscopic examination, histopathologic study, cytopathologic study, culture from sterile material, blood culture, tissue nucleic acid diagnosis, or serological tests. All patients diagnosed with myositis, enthesitis, bursitis, septic arthritis, or osteomyelitis from fungal infection were identified using the following International Classification of Diseases (ICD)-10 codes for principle diagnosis and comorbidity: B49 (fungus), B378 (Candida (candidiasis)), B453 (Cryptococcus (cryptococcosis) and Osseous cryptococcus), B393, B399 (Histoplasma (histoplasmosis)), B388 (Coccidioides (coccidioidomycosis)), B428 (Sporothrix (Sporothrix schenckii) and sporotrichosis), B484 (Penicillium (penicillosis) and Talaromyces), B59 (Pneumocystis (pneumocystosis)), B468 (Mucor (mucormycosis)), M902 (osteomyelitis/bone), M0164 (arthritis (B35-B49)), M688 (tendinitis/tendonitis/enthesitis), M738 (bursitis), and M632 (myositis (B35-B49)). The protocol for this study was approved by the Siriraj Institutional Review Board (SIRB) (COA no. Si 731/2020). Written informed consent was not obtained from the included patients due to our study’s anonymous retrospective design.

Demographic, extra-articular fungal infection, underlying joint disease, comorbidity, concomitant medication, onset of infection, mechanism of musculoskeletal infection, clinical manifestation, laboratory finding, histopathologic finding, radiologic finding, treatment, and outcome data were collected for each included patient.

Regarding the onset of infection [4], ‘breakthrough infection’ was defined as the development of infection in a patient who was already receiving one or more mold-active antifungal agents at the clinically apparent onset of diagnosis, while ‘de novo infection’ was defined as the development of infection in a patient who was not receiving a mold-active antifungal agent at the clinically apparent onset of infection or during the previous three days.

The mechanisms of musculoskeletal infection were stratified into direct inoculation, hematogenous spreading, or contiguous spreading. Direct inoculation was defined as the seeding of musculoskeletal tissue by trauma or surgical manipulation. Hematogenous spreading was defined as the seeding of musculoskeletal tissue by the blood-borne route. Contiguous spreading was defined as the seeding of musculoskeletal tissue from an adjacent focus of infection. For treatment outcomes [4], ‘complete response’ (CR) was defined as the complete resolution of clinical and radiologic findings at the sites of infection, and ‘partial response’ (PR) was defined as the partial resolution of clinical and/or radiologic findings at the sites of infection or partial clinical improvement without the availability of radiologic data.

### Statistical Analysis

Patient demographic and clinical data were summarized using descriptive statistics. The categorical data are presented as the number and percentage, and the continuous data are presented as the mean plus/minus the standard deviation for normally distributed data, or as the median and range for non-normally distributed data. There was no extrapolation for missing data. The statistical analyses were performed using PASW Statistics for Windows (version 18.0; SPSS, Inc., Chicago, IL, USA).

## 3. Results

The search of our center’s medical record database using the aforementioned ICD-10 codes directly or indirectly related to musculoskeletal fungal infection yielded 185 potential cases; however, only 28 of those cases met our inclusion criteria. There were 22 cases (78.5%) of fungal osteomyelitis, 1 case (3.6%) of septic arthritis, and 5 cases (17.9%) of coexisting fungal osteomyelitis and septic arthritis (Table S1).

Musculoskeletal fungal infection occurred slightly more frequently in males (57.1% vs. 42.9%). The median age among all included patients was 58.5 years (range: 22–81), and the median body mass index (BMI) was 21.96 kg/m^2^ (range: 15–27). Almost half were unemployed (42.9%), 21.4% were farmers, 17.9% were general employees, 3.6% were employed as a police officer, a nurse, a grocer, and a factory worker, and one case had an unidentified occupation.

The majority of patients (82.1%) had underlying diseases that adversely affected their systemic immunity and/or local immune defense, including chronic kidney disease (CKD) (75%), overweight status (39.3%), diabetes mellitus (DM) (35.7%), current smoker (39.3%), adult-onset immunodeficiency with anti-interferon gamma autoantibody (21.4%), alcohol use (21.4%), asthma with allergic rhinitis (7.1%), underweight status (10.7%), cirrhosis (7.1%), malignancy (3.6%), thalassemia (3.6%), and an indwelling long-term catheter (3.6%). Additionally, 14.3% had previous local musculoskeletal and soft tissue problems, including previous septic arthritis and/or osteomyelitis (3.6%), prosthetic joints (7.1%), and previous superficial skin infection at the affected area (3.6%). No included patient had HIV infection, neuropathy, previous radiation at the affected bone area, peripheral vascular disease, or venous stasis.

All cases were definitively diagnosed as having fungal infection via the identification of the organism(s) from a direct culture and/or histopathologic study. All infections were de novo infections, except for 3.6% of cases, which were breakthrough infections. Regarding the mechanism of infection, hematogenous spreading was the most common route (75%), followed by direct inoculation (25%).

### 3.1. Clinical Manifestations

Local symptoms were common. Pain and tenderness, which were the most common manifestations of musculoskeletal fungal infection, occurred in 82.1% of cases. Other symptoms included swollen lesions (71.4%), soft tissue invasion (35.7%), and abscess formation (32.1%). In the musculoskeletal fungal infection of the skull and sinuses, the local symptoms included headaches (3.6%), dental pain (3.6%), and nose tightness (3.6%). Symptom onset tended to be gradual, and the median duration of symptoms was 2 months (range: 0.5–120). There was one case of chronic osteomyelitis at a metatarsal bone with *Paecilomyces* spp. infection that presented with a chronic foot ulcer for 10 years prior to diagnosis.

More than half of the subjects had systemic symptoms (60.7%), including fever (39.3%), pulmonary infiltration (17.9%), and skin rash (17.9%). Skin rashes were characterized by generalized papules and/or nodules. All cases with pulmonary infiltration and skin lesions had *Taralomycosis marneffei* infection.

### 3.2. Articular Distribution

All patients presented single, non-contiguously infected sites, except for two patients who had an infection at more than one site. One patient had septic arthritis of the hip and knee, and another had *T*. *marneffei* fungemia with disseminated musculoskeletal fungal infection at the skull, right 4th, 7th, 8th, and 10th ribs, right humerus, right radius, both ulnar bones, left acetabulum, both femurs, and both tibias.

Regarding the site of musculoskeletal fungal infection, a total of 40 sites were reported, including 33 bony lesions (82.5%) and 7 joint lesions (17.5%). Among those with osteomyelitis, the tibia was the most common site (30%), followed by the foot (9%), skull (9%), hand (6%), rib (6%), humerus (6%), ulna (6%), femur (6%), sinus (6%), spine (3%), scapula (3%), clavicle (3%), radius (3%), and acetabulum (3%). Concerning joint lesions, the knee was the most commonly involved joint (72%), followed by the shoulder (14%) and hip (14%). In patients with septic arthritis (n = 6), adjacent osteomyelitis was found in 67%. The most common site of adjacent osteomyelitis with septic arthritis was the tibia with septic arthritis at the knee (50%), followed by osteomyelitis at the humerus with septic arthritis at the shoulder (17%).

### 3.3. Diagnosis

Diagnosis of musculoskeletal fungal infection was most frequently established via tissue biopsy. Specimens were sent to identify the pathogen(s) by direct culture for fungus (100%) and/or histopathology (92.86%), which yielded positive result rates of 100% and 57.69%, respectively. Among those with septic arthritis (n = 6), synovial fluid analysis was performed in four cases, and fungal septic arthritis was diagnosed by direct fungal culture in two cases. Histopathologic studies were performed in five cases. The most commonly reported findings were non-specific synovitis, chronic inflammation, and/or granulomatous formation.

Standard culture from specimens was performed in all cases, and 36.4% showed evidence of coexisting fungal and bacterial infection. Hemoculture for aerobic bacteria was performed in all cases, and none had a positive result. Of the 60.7% of patients who underwent hemoculture, fungi and fungemia were detected in 17.9%. All cases of fungemia were caused by *T. marneffei*.

DNA testing and serological studies were conducted only in four cases. DNA testing was performed in three cases, and only one of those cases was positive for *Rhizopus oryzae* in a patient with invasive fungal sinusitis. Regarding the serological studies, serum cryptococcal antigen was positive in a patient with cryptococcal osteomyelitis of the phalangeal bone.

### 3.4. Microbiology

The most commonly identified pathogen was *T*. *marneffei* (28.6%), followed by *Cryptococcus neoformans* (17.9%), *Aspergillus* spp. (10.7%), *Candida parasilopsis* (10.7%), *Fusarium* spp. (14.3%), *Lomentospora prolificans* (3.6%), *Paecilomyces* spp. (3.6%), *R. oryzae* (3.6%), *Scedosporium apiospermum* (3.6%), and an unidentified hyaline septate mold (3.6%).

Among patients with fungal and bacterial coinfection identified from tissue specimens (8/28), *Staphylococcus* spp. was the most common organism (50%), followed by Gram-negative bacilli, including *Pseudomonas aeruginosa* (25%), *Escherichia coli* (12.5%), and *Klebsiella pneumoniae* (12.5%).

### 3.5. Histopathologic Profiles

Histopathologic studies were performed in 93% of all specimens from the affected sites. Most reports described acute and chronic inflammation with or without a granuloma. Fungi with their characteristic morphology were also described (Table 1).

### 3.6. Blood Chemistry

The complete blood count (CBC) and blood chemistry results were non-specific. CBC demonstrated mild anemia (median hemoglobin: 9 g/dL (range: 6–13)), a normal white blood cell (WBC) count (median WBC count: 8850 cells/ul (range: 2900–23,600)), median neutrophils (median neutrophil concentration: 67% (range: 43–91)), and a normal platelet (PT) count (median PT count: 316.5 × 10^9^/L (range: 133–859)). The median estimated glomerular filtration rate (eGFR) was 59.5 mL/min/1.73 m^2^ (range: 12–324), the median serum albumin level was below the normal range (2.9 g/dL (range: 1.7–4.6)), and there was no report of abnormal liver enzymes.

Serum assays for the erythrocyte sedimentation rate (ESR) and C-reactive protein (CRP) were sent in 57% and 75% of cases, respectively. The results demonstrated high values for these inflammatory markers (median ESR: 63.5 mm/h (range: 28–119), and median CRP: 31.44 mg/L (range: 4.18–223)).

### 3.7. Diagnostic Imaging

*Plain radiography* was performed in 75% of all cases. The most common findings were well-defined osteomyelitic lesions, narrowing of the joint space, contagious bone destruction with periarticular osteopenia, and soft tissue swelling.

*Computed tomography* was performed in 25% of cases. The most common findings were bony destruction and ill-defined soft tissue swelling or mass with enhancement.

*Magnetic resonance imaging* was conducted in 27.3% of cases. The most common finding was additional detail relating to soft tissue invasion, including mass, sinus tract, torn tendon, bony destruction, and bone marrow edema.

*Bone scan* was conducted in 17.9% of cases. Almost half (40%) of patients who underwent bone scans had clinical suspicion of multiple sites of musculoskeletal fungal infection.

### 3.8. Treatments and Outcomes

All patients were treated with systemic antifungal agents, including voriconazole (25%), fluconazole (10.7%), amphotericin B (10.7%), itraconazole (10.7%), micafungin (7.1%), amphotericin B switched to fluconazole (7.1%), amphotericin B switched to itraconazole (14.4%), amphotericin B switched to voriconazole (7.1%), amphotericin B switched to voriconazole and later itraconazole (3.6%), and itraconazole switched to voriconazole (3.6%). Twenty-six of 28 patients (82.3%) received surgical management in addition to systemic antifungal therapy. The median duration of antifungal therapy was 12 months (range: 0.5–21). The longest duration of treatment was given to a patient with disseminated *T*. *maneffei* infection. Surgical intervention was required for both diagnosis and local infection control.

All included patients were hospitalized at first diagnosis. The median length of hospital stay was 20 days (range: 1–75). Major complications included primary bacteremia (one case), septic shock with multiple organ damage (one case), and antifungal agent-induced liver injury (two cases).

At the end of treatment, the proportions of patients with CR and PR were 50% and 32%, respectively. The treatment response was unknown in 18% of patients due to either loss to follow-up or missing/incomplete data in the medical record. All patients with CR had combination treatment with systemic antifungal therapy and surgical management. Among the patients with PR, 71.4% received combination therapy and 28.6% received systemic antifungal therapy alone.

## 4. Discussion

This study comprehensively reviewed and analyzed cases diagnosed with musculoskeletal fungal infection by various types of fungal species to identify the key features of musculoskeletal fungal infection that are not sufficiently well understood based only on previous case series or individual case reports. In this study, we report additional details related to all cases of musculoskeletal fungal infection that were diagnosed and treated at our national tertiary referral center over the last two decades.

Similar to previous studies, we found musculoskeletal fungal infection to be more common in males. The median age (58.5 years) in our study was older than the median/average age reported in other studies (range: 38.2–50 years) [5,6,7]. Consistent with previous studies, the majority of the patients in our study had underlying diseases that adversely influence immune responses, such as diabetes and alcoholism [3,5,8]. However, some risk factors reported in other studies, including prior use of broad-spectrum antibiotics/antifungal agents [6], immunosuppressant administration, chronic granulomatous disease, organ transplantation [4,7], and HIV infection [3], were not discovered in our study’s small sample size.

Concerning clinical characteristics, our findings correspond with those from previous studies in many aspects. Pain and tenderness were the most common local manifestations [4,6,7], whereas fever presented in less than half of the cases [4,6,7]. In addition, most infections were de novo, and the main mechanism of infection was hematogenous spreading [4,6,7]. The duration of symptoms varied, and the onset of symptoms was gradual. We also found durations of symptoms prior to diagnosis of up to 10 years.

In contrast to other studies that reported musculoskeletal fungal infection predominance at the vertebrae [4,6], we found the tibia, foot, and skull to be most often affected. All musculoskeletal fungal infections caused by yeasts occurred at the lower extremities, except for one case of cryptococcosis that affected the hand. We also found that Aspergillus osteomyelitis originated from the paranasal sinuses, and the knee was the most commonly affected joint, which is similar to other studies [2,7]. Interestingly, our study found that fungal septic arthritis usually coexisted with adjacent bone osteomyelitis, and the most common coinfections in this study were osteomyelitis at the tibia and septic arthritis at the knee. Based on this finding, once fungal septic arthritis is established, investigation for the presence of adjacent bone osteomyelitis should be performed because surgical debridement and a longer duration of systemic antifungal therapy may be required.

Most previous studies were case reports or case series of various organisms, so the common causative pathogens among musculoskeletal fungal infection were not investigated. Our study is the first to describe the three most common fungal pathogens in the musculoskeletal system, which were found to be *T. marneffei*, *C*. *neoformans,* and *Fusarium* spp., consecutively. This finding is compatible with previous studies, indicating that *T. marneffei* infection is endemic in tropical Asia, especially Thailand, northeastern India, China, Hong Kong, Vietnam, and Taiwan [9].

For the diagnosis of musculoskeletal fungal infection, organism identification was essential because musculoskeletal fungal infection was found to lack specificity in clinical manifestations, blood chemistry, and radiographic findings. Similar to previous studies, our results showed the WBC count in the complete blood count to be in the normal range [4,6,7].

Regarding the elevation of inflammatory markers, our findings are similar to those of previous studies that reported a mean ESR level of 86 mm/h and a mean CRP level of 51.5 mg/L in *Aspergillus* osteomyelitis [4]. These findings suggest that musculoskeletal fungal infection affects systemic inflammation activity. However, the elevation of inflammatory markers is not specific to musculoskeletal fungal infection. Inflammatory markers may also be elevated in other conditions, including endocarditis; pharyngitis; vascular graft infections; rheumatologic diseases, such as crystal-induced arthritis; malignancy; and drug reactions [10]. Nevertheless, ESR and CRP are helpful tools for monitoring responses to treatment. A previous study reported that the absence of elevated serum WBC, ESR, or CRP did not exclude the diagnosis of septic arthritis [11]. Therefore, the diagnosis of musculoskeletal fungal infection requires tissue for organism identification. We found that surgical biopsy for tissue culture and histopathologic studies to be the most reliable method for diagnosing musculoskeletal fungal infection.

Although imaging studies do not provide pathogen-specific information, they were found to be useful for detecting the nature and extension of musculoskeletal fungal infection. Plain musculoskeletal radiography was the most frequent imaging study used, and it could well detect osteomyelitic lesions. Computed tomography was helpful for identifying bony destruction and ill-defined soft tissue swelling or masses with enhancement. Magnetic resonance imaging provided additional detail relating to soft tissue invasion, including mass, sinus tract, torn tendon, bony destruction, and bone marrow edema. Bone scans were commonly ordered in patients with clinical suspicion of multiple sites of musculoskeletal fungal infection.

Regarding treatment, most patients in our study required surgical management in addition to systemic antifungal therapy, with a median duration of antifungal therapy of 12 months (range: 0.5–21). Regarding the treatment of *Candida* osteomyelitis in a previous study, combination therapy was administered in 48% of cases, with a median duration of treatment of 90 days (range: 7–720) [6]. In another study that reported the treatment of *Aspergillus* osteomyelitis, combination therapy was administered for 67% of patients, with a median duration of treatment of 90 days (range: 10–772) [4]. The response to treatment in the present study was somewhat different from that in other reports. The rate of CR in our study was higher than the previously reported CR rate among patients with Candida osteomyelitis of 32% [6]. In contrast, our CR and PR rates were similar to the CR rate of 57% and PR rate of 30% reported by a study on Aspergillus osteomyelitis [4]. These variations may reflect differences in disease severity that are influenced by the host setting, causative organisms, and site of infection, as well as combination surgery and systemic antifungal therapy, which influenced a higher response rate.

This study has some mentionable limitations. First, our study’s retrospective design rendered it vulnerable to missing or incomplete data. Second, due to the relative rarity of musculoskeletal fungal infection, only 28 cases were enrolled during the almost 20-year study period. Third, our data were from a single center, which also happened to be Thailand’s largest national tertiary referral center. This fact has two notable implications. First, since our center is routinely referred complicated cases that are thought to be untreatable in other medical care settings, our scarcity of cases supports the rarity of this disease in Thailand. Second, our findings may not be generalizable to other care settings in Thailand. A systematic review and meta-analysis of cases and case series reported in Thailand may be needed to better understand the characteristics and outcomes of this disease in Thailand. 

In conclusion, musculoskeletal fungal infection is a rare disease in Thailand, and fungal osteomyelitis was found to be the most prevalent type of fungal infection. This disease is characterized by insidious onset and non-specific manifestation, so pathogen identification via tissue culture and histopathologic studies is required. Imaging studies provide additional information regarding disease extension and severity. Surgical management in combination with antifungal therapy was required in most cases, and the outcome of treatment was generally favorable.

## Figures and Tables

**Table 1:** Pathogen, site of infection, type of infection, and histopathologic findings in patients with fungal musculoskeletal infection.

Pathogen	Site of Infection	Type of Infection	Histopathologic Report
*Aspergillus flavus*	Skull	Osteomyelitis	Dichotomous branching septate hyphae
*Aspergillus fumigatus*	Skull	Osteomyelitis	Acute and chronic inflammation with presence of non-pigmented septate fungal hyphae in sinus, orbital tissue, and bone
*Aspergillus* spp.	Rib	Osteomyelitis	Suppurative granulomatous inflammation
*Candida parapsilosis*	Knee	Septic arthritis	Prosthetic membrane with moderate acute and chronic inflammation and histiocyte aggregation
*Candida parapsilosis*	Hip and knee	Septic arthritis	Nonspecific synovitis, negative for granuloma and malignancy
*Candida parapsilosis*	Tibia	Osteomyelitis	Prosthetic membrane with moderate acute and chronic inflammation and histiocyte aggregation
*Cryptococcus neoformans*	Hand	Osteomyelitis	Not available
*Cryptococcus neoformans*	Tibia	Osteomyelitis	Budding yeast osteomyelitis with B cell lymphoid neoplasm
*Cryptococcus neoformans*	Tibia	Osteomyelitis	Chronic osteomyelitis with fungal-associated chronic granulomatous inflammation suggestive of cryptococcosis
*Cryptococcus neoformans*	Tibia	Osteomyelitis	Cryptococcus osteomyelitis
*Cryptococcus neoformans*	Tibia	Osteomyelitis	Fungal-associated osteomyelitis
*Fusarium* spp.	Foot	Osteomyelitis	Acute suppurative inflammation with granulomatous reaction, Gomori methenamine silver stain reviewed broad branching septate hyphae compatible with fusarium
*Fusarium* spp.	Tibia	Osteomyelitis	Branching septate hyphae suggestive of *Fusarium* spp.
*Fusarium* spp.	Tibia	Osteomyelitis	Acute suppurative inflammation with granulomatous reaction, Gomori methenamine silver stain reviewed broad branching septate hyphae compatible with fusarium
*Lomentospora prolificans*	Spine	Osteomyelitis	Focal necrotizing granuloma with presence of generated branching septate fungal hyphae
*Paecilomyces* spp.	Foot	Osteomyelitis	Soft tissue with histiocyte aggregation and mild chronic inflammation
*Rhizopus oryzae*	Sinus	Osteomyelitis	Degenerated fungal hyphae with non-pigmentation and rare septation, invasive fungal infection involving maxillary sinus
*Scedosporium apiospermum*	Foot	Osteomyelitis	Eumycotic mycetoma due to hyaline fungi
*Talaromyces marneffei*	Hand	Osteomyelitis	Mixed follicular, paracortical hyperplasia, focal capsular thickening with marked plasmacytic infiltration and admixed neutrophils, eosinophiles, and histiocytes, negative for bacteria, acid-fast bacilli, spirocete, and fungi
*Talaromyces marneffei*	Humerus	Osteomyelitis	Acute and chronic inflammation with granuloma tissue formation and xanthomatous reaction
*Talaromyces marneffei*	Knee	Septic arthritis	Not available
*Talaromyces marneffei*	Knee	Septic arthritis	Negative for organism, acute osteomyelitis and acute synovitis
*Talaromyces marneffei*	Scapula and clavicle	Osteomyelitis	Chronic ulcer and granulation tissue with acute and chronic granuloma, presence of small yeast-formed fungi
*Talaromyces marneffei*	Shoulder	Septic arthritis	Acute and chronic inflammation with granuloma tissue formation and xanthomatous reaction
*Talaromyces marneffei*	Tibia	Osteomyelitis	Negative for organism
*Talaromyces marneffei*	Disseminated infection, including radius bone	Osteomyelitis	Chronic osteomyelitis
Unidentified hyaline septate mold	Sinus	Osteomyelitis	Scattered degenerated branching septate hyphae

## Data Availability

The data presented in this study are available in supplement material (Table S1).

## Supplementary Materials

The following supporting information can be downloaded at: https://www.mdpi.com/article/10.3390/jof8020191/s1, Table S1: Summary of detailed clinical data of 28 patients with MSK fungal infection.
