# Supplementary material for: Risk Factors, Clinical Characteristics, Management, and Outcomes of Musculoskeletal Fungal Infection at Thailand’s Largest National Tertiary Referral Center
Source: J Fungi (Basel). 2022 Feb 16;8(2):191. doi: 10.3390/jof8020191 (PMC8880593; doi:10.3390/jof8020191)
Supplement: Supplementary file 1 [file jof-08-00191-s001.zip › jof-1575523-supplementary.pdf]

Supplementary Table S1. Summary of detailed clinical data of 28 patients with MSK fungal infection.

Age (Year)	Sex	Underlying Disease	Pathogen	Site of Infection	Type of Infection	Symptom Duration (Month)	Mechanism of Infection	Antifungal Treatment	Duration of Antifungal Treatment (Month)	Surgery	Treatment Response
22	Male	Overweight, alcohol drinking, smoking	*T .marneffei*	Scapular clavicle	Osteomyelitis	2	Hematogenous	Amphotericin B	N/A	Yes	N/A
22	Male	Overweight, alcohol drinking, smoking	*T .marneffei*	Knee	Septic arthritis	2	Hematogenous	Amphotericin B	N/A	Yes	N/A
34	Female	Cirrhosis CTP B, underweight, DM, ESRD on indwelling long term catheter, thalassemia	*C .parasilopsis*	Hip Knee	Septic arthritis	9	Hematogenous	Amphotericin B, fluconazole,	0.5	Yes	PR
44	Male	Overweight, alcohol drinking, smoking	*Scadeosporium apiosperum*	Foot	Osteomyelitis	12	Direct inoculation	Voriconazole	9	Yes	CR
45	Female	CKD stage 2	*C .neoforman*	Tibia	Osteomyelitis	6	Hematogenous	Fluconazole	12	Yes	CR
46	Female	CKD stage 2	*C .neoforman*	Tibia	Osteomyelitis	6	Hematogenous	fluconazole	12	Yes	CR
47	Female	DM, CKD stage 2	*T .marneffei*	Disseminated infection		0.5	Hematogenous	Amphotericin B, itraconazole	21	No	PR
51	Male	CKD stage 2, AOID with IGA	*T .marneffei*	Knee	Septic arthritis	0.5	Hematogenous	Itraconazole	12	Yes	CR
51	Male	CKD stage 2, AOID with IGA	*T .marneffei*	Tibia	Osteomyelitis	0.5	Hematogenous	Itraconazole	12	Yes	CR
53	Female	CKD stage 4, AOID with IGA	*T .marneffei*	Humerus	Osteomyelitis	1	Hematogenous	Amphotericin B, itraconazole	13	Yes	PR
53	Female	CKD stage 4, Cirrhosis CTP C, AOID with IGA	*T .marneffei*	Shoulder	Septic arthritis	1	Hematogenous	Amphotericin B, itraconazole	13	Yes	PR
54	Male	DM, CKD stage 3a, overweight, alcohol drinking	*Fusarium spp.*	Foot	Osteomyelitis	3	Hematogenous	Voriconazole	12	Yes	CR

54	Female	Overweight	*Lomentospora prolificans*	Vertebra	Osteomyelitis	12	Hematogenous	Amphotericin B	N/A	No	N/A
58	Female	AOID with IGA	*C .neoforman*	Hand	Osteomyelitis	0.5	Hematogenous	Amphotericin B, fluconazole	13	Yes	CR
59	Male	DM, CKD stage 4, overweight, smoking	*Aspergillus fumigatus*	Skull	Osteomyelitis	2	Direct inoculation	Amphotericin B, voriconazole	7	Yes	PR
62	Male	CKD stage 4	*Aspergillus spp.*	Rib	Osteomyelitis	7	Hematogenous	Voriconazole	3	Yes	CR
63	Female	DM, overweight, asthma and allergic rhinitis	*Rhizopus oryzae*	Sinus	Osteomyelitis	0.5	Direct inoculation	Voriconazole	2	Yes	CR
63	Female	DM, overweight, asthma and allergic rhinitis	Unidentified hyaline septate mold	Sinus	Osteomyelitis	1	Direct inoculation	Amphotericin B, voriconazole, itraconazole	13	Yes	PR
67	Male	CKD stage 3a, overweight	*Paecilomyces spp.*	Foot	Osteomyelitis	120	Hematogenous	Itraconazole, voriconazole	9	No	PR
70	Male	CKD stage 3b, overweight, alcohol drinking, smoking	*Candida parasilopsis*	Knee	Septic arthritis	2	Direct inoculation	Micafungin	7	Yes	PR
70	Male	CKD stage 3b, overweight, alcohol drinking, smoking	*Candida parasilopsis*	Tibia	Osteomyelitis	2	Direct inoculation	Micafungin	7	Yes	PR
71	Male	DM, CKD stage 3b, smoking	*Fusarium spp.*	Knee	Septic arthritis	2	Hematogenous	Voriconazole	7	Yes	CR
71	Male	DM, CKD stage 3b, smoking	*Fusarium spp.*	Tibia	Osteomyelitis	2	Hematogenous	Voriconazole	7	Yes	CR
72	Male	DM, CKD stage 3b, underweight, smoking	*Fusarium spp.*	Tibia	Osteomyelitis	2	Hematogenous	Voriconazole	7	Yes	CR
74	Female	CKD stage 3b, AOID with IGA	*T .marneffei*	Hand	Osteomyelitis	2	Hematogenous	Amphotericin B, itraconazole	0.25	Yes	CR
76	Female	DM, CKD stage 4	*Aspergillus flavus*	Skull	Osteomyelitis	3	Direct inoculation	Amphotericin B, voriconazole	6.5	Yes	CR
80	Male	CKD stage 3a	*C .neoforman*	Tibia	Osteomyelitis	3	Hematogenous	Fluconazole	16	Yes	N/A
81	Male	CKD stage 2, lymphoma	*C .neoforman*	Tibia	Osteomyelitis	3	Hematogenous	Itraconazole	12	Yes	N/A

AOID with IGA, adult-onset immunodeficiency with anti–IFN-γ autoantibodies; BMI, body mass index; CKD, chronic kidney disease; CR, complete response; CTP, Child–Turcotte–Pugh score; DM, diabetes mellitus; ESRD, end-stage renal disease; PR, partial response; N/A, not available.
